# Detecting presence of cardiovascular disease through mitochondria respiration as depicted through biophotonic emission

**DOI:** 10.1016/j.redox.2015.11.014

**Published:** 2015-11-29

**Authors:** Nancy R. Rizzo, Nicole C. Hank, Jian Zhang

**Affiliations:** a EPIC Research & Diagnostics, Inc., 7659 E. Pinnacle Peak Road, Suite 115, Scottsdale, AZ 85255, USA

**Keywords:** Mitochondria, Cardiovascular disease, Biophotons, Reactive oxidative stress (ROS), Clearview System

## Abstract

**Aims:**

Increased production of reactive oxygen species (ROS) in mitochondria, play an important role in the cardiovascular system. Furthermore, oxidative metabolism of mitochondria comprised of biophoton emissions, are linked to ROS and oxidative stress. In this review we investigated the association between the ability of ClearViewTM system (ClearView) to indicate the presence or absence of cardiovascular disease through mitochondria respiration as depicted through biophotonic emission.

**Methods and results:**

One hundred and ninety-five out of the three hundred and fifty-three human subjects enrolled in this prospective, single site study had at least one cardiovascular related diagnosis. Measurements with ClearView consisted of scanning all 10 fingers twice. Images were captured through the ClearView software and analyzed to produce a scale that indicates the presence or absence of cardiovascular disease. The association of ClearView's ability to indicate the presence or absence of cardiovascular disease with a physician's diagnosis was assessed using odds ratios (OR) and area under ROC curve (AUC). Adjusting for age, OR of ClearView measurements conducted with capacitive barrier was 3.44 (95%CI: 2.13, 5.55) and the OR without the capacitive barrier was 2.15 (95%CI: 1.42, 3.23). The OR in men were 5.91 (95%CI: 2.35, 14.85) and 2.88 (95%CI: 1.38, 6.01), adjusting for age and corresponding to with and without capacitive barrier. The OR in women were 3.50 (95%CI: 1.86, 6.59) and 2.09 (95%CI: 1.20, 3.64) with and without capacitive barrier. AUCs for measurements with capacitive barrier were >0.90.

**Conclusion:**

ClearView detected the presence or absence of cardiovascular disease independent of other conditions.

## Highlights

• Detecting components of cardiovascular disease through biophoton emission.
• Non-invasive way of measuring in-vivo cell to cell communication.
• Electrophysiological tool detects presence or absence of cardiovascular disease.

## Graphical abstract

**Figure:**
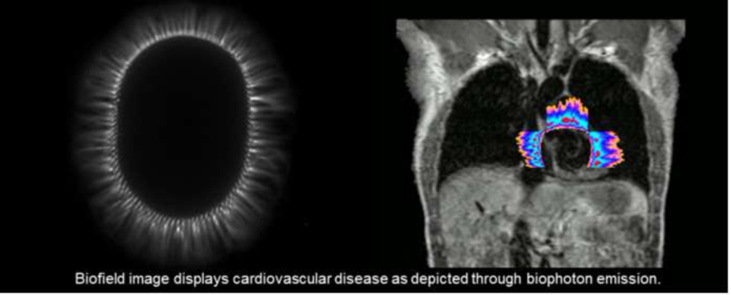

## 1 Introduction

Cardiovascular diseases (CVD), including hypertension, coronary artery disease (CAD), cardiomyopathy, heart failure, and stroke are the greatest cause of mortality, with over 17.3 million people dying annually [1],costing $863 billion in direct and indirect economic expenditures each year [2]. Cardiovascular disease is a common complex disorder, which can be caused by a single gene or multifactorial conditions resulting from interactions between environmental (modifiable) and inherited (immutable) risk factors [3]. While significant progress in understanding the development of CVD has been discovered, the mechanisms of individual CVD susceptibility are still not clearly understood. A considerable number of studies suggest that altered levels of oxidative stress within the cardiovascular environment are critical contributors in the development of cardiovascular disease [4] while others suggest CVD is a multifactorial disorder that involves several genetic determinants, including mitochondrial dysfunction [5]. Mitochondrial dysfunction has been associated with a wide range of cardiovascular disorders such as cardiomyopathy and hypertension [6]. In this regard, studies are beginning to illustrate that mitochondria not only appear susceptible to damage mediated by increased oxidative stress but also play a significant role in the regulation of cardiovascular cell function [4]. Furthermore, oxidative metabolism of the mitochondria via biophoton emission, is linked to increased production of reactive oxygen species (ROS) and overall oxidative stress. This has led to accumulating evidence that a commonality among cardiovascular disease development and cardiovascular disease risk factors is due to increased mitochondrial damage and dysfunction [4] which can be measured through biophotonic emission. In this manuscript we will introduce the utilization of the Clearview System (ClearView), which can detect cardiovascular disease through the measurement of mitochondria dysfunction through biophoton emission.

### 1.1 Mitochondrial dysfunction associated with CVD

**Fig. 1:**
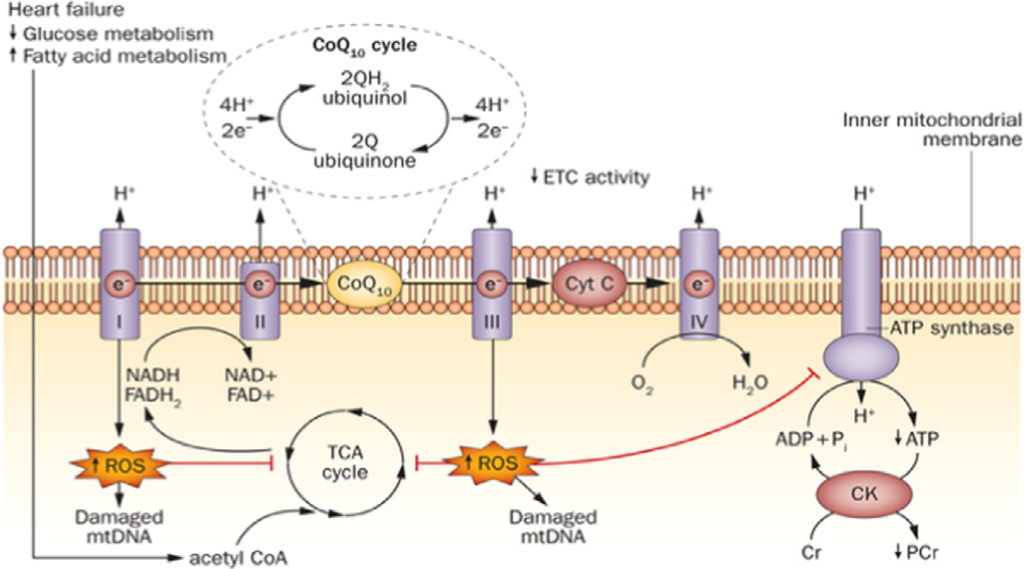
Mitochondrial dysfunction and oxidative stress in heart failure [21].

Mitochondria, also referred to as the “powerhouses” of cells, are responsible for creating over 90% of energy needed by the body to sustain life and support growth [7]. Not only do they generate energy in the form of ATP, but they also regulate numerous cellular functions relevant to cell outcome, such as apoptosis, generation of oxidative free radicals, and calcium homeostasis. Mitochondria are a major source of reactive oxygen species (ROS). Oxidative stress occurs when excess ROS are generated and cannot be adequately countered by the antioxidant systems [8]. Oxidative stress has also been associated with loss of cells in heart failure, cardiomyopathy, and increased ROS associated with mitochondrial dysfunction in heart failure [8], suggesting there is a strong affiliation between CVD development, and mitochondrial damage and function [9] (Fig. 1).

Several factors can increase the risk of CVD; however, there is lack of clarity to whether the risk factors alter cellular function. Studies have demonstrated that several CVD risk factors such as hypercholesterolemia and age can alter mitochondrial damage and function [10] by increasing mitochondrial oxidative stress. It has also been thought that CVD risk factors may act on the cardio-vasculature by inducing mitochondrial damage and dysfunction, thus facilitating a premature aging process [11]. Various studies are focused on identifying mitochondrial sources of ROS; however, these are limited to in vitro studies and lack the ability to detect possible disruption of cellular communication as a contributor. Through oxidative metabolism of the mitochondria exhibited by biophoton emissions, we will demonstrate the ClearView system's capability of detecting and measuring cell-to-cell communication in vivo.

### 1.2 Biophoton detection

**Fig. 2:**
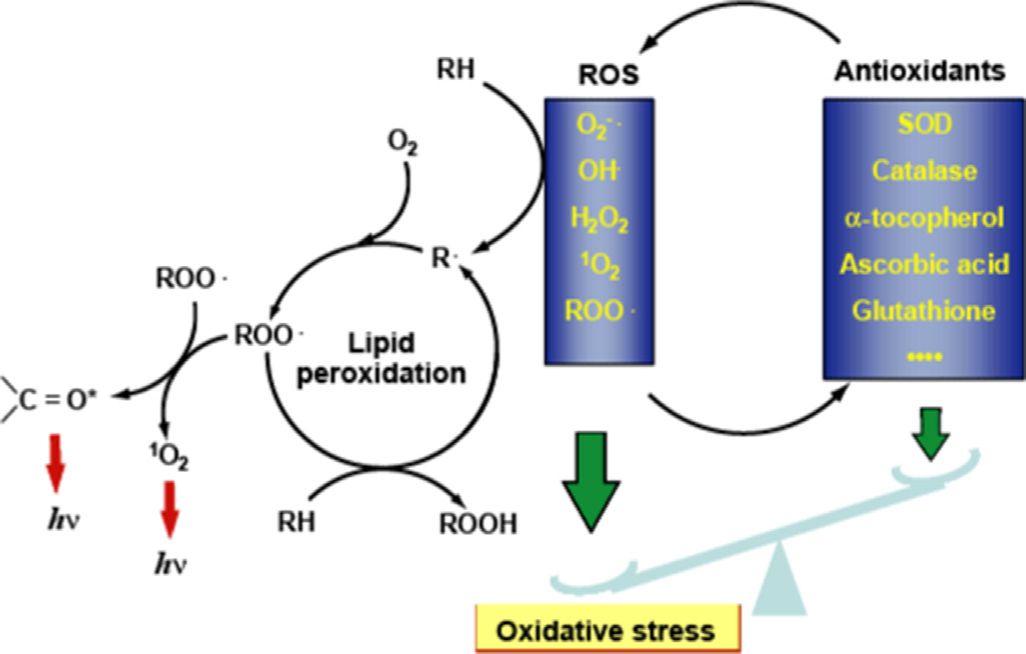
Illustration of biophoton mechanism [22].

Biophoton emission, the spontaneous emission of light (photons) emanating from all living systems including humans, is biochemically distinct from bioluminescence, which is generally visible and involves specialized enzymatic mechanisms. Although biophoton emission is described to be less than 1000 photons per second per cm and are not visible to the naked eye [12], recent advances in photo-detection make it possible to analyze biophoton emission. Biophoton emission is found in oxidative metabolism in mitochondria, free radical reactions with biomolecules, proteins, and DNA. Biophoton emission has been known to be related to the generation of ROS, and therefore could be used as a tool for the investigation of oxidative stress [13] (Fig. 2). Boveris and Cadenas [14] were the first to demonstrate the potential usefulness of biophoton detection for non-invasive monitoring of oxidative metabolism and oxidative damage in living tissue. Several studies repeatedly illustrate that the intensity of photon emission changes in a state of disease [15] and that disease cells emit significantly more biophotons than healthy cells. It has also been shown that changes in biophotonic activity are indicative of changes in mitochondrial ATP energy production manifested in physiological and pathological conditions [16]. Biophoton emission has also been found to be an expression of cell-to-cell communication reflective of the functional state of the living organism, and its measurement can be used to assess this state [17]. Over decades of research in this area, several studies have made progress in investigating human biophoton emission in both basic and applied research. Clinically relevant in vivo human biophoton detection is currently being studied; however, the numbers of studies and human subjects have been limited. Although the use of biophoton emission for diagnostic and treatment purposes is in its infancy, we have demonstrated the ability to detect several components of cardiovascular disease through biophoton emission as assessed by the ClearView system.

### 1.3 ClearView system

The ClearView system is a non-invasive, electrophysiological measurement tool that has the possibility to determine autonomic response and physical response indicators by measuring the electrophysiological signals associated with human body systems. It has been scientifically proven that every cell in the body emits more than 100,000 light impulses or photons per second. These light emissions, otherwise known as biophotons, have been found to be the steering mechanism behind all biochemical reactions. Unlike other bio-impedance devices, such as the electrocardiogram (ECG) and the electroencephalogram (EEG), that measures the electrophysiology of the heart and brain, the ClearView system measures electromagnetic energy at a smaller scale through amplification of biophotons. It has been demonstrated that biophoton emission is strongest in the hands [18]. It has also been demonstrated that if a disease is present, biophotonic imbalances are emitted between left and right hands, suggesting diagnostic potential [19]. The ClearView system taps into the global electromagnetic holographic communication system via the fingertips. The fingers are a highly refined component of the peripheral nervous system; therefore, due to the high energy expenditures of the nerves throughout the body, they require higher proportionate numbers of mitochondria. Through measuring mitochondrial respiration via biophoton detection, the ClearView system has the ability to quantify electrophysiological biophoton activity as it relates to the metabolic function of forty-nine (49) identified organ systems.

**Fig. 3:**
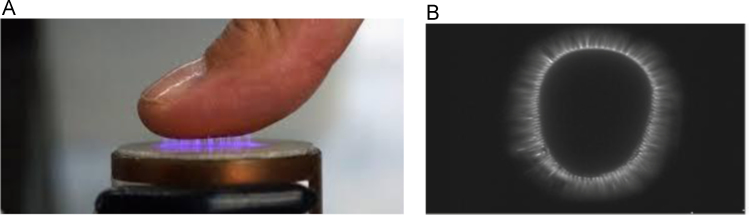
(A) High frequency voltage impulse generating localized electromagnetic field. (B) Energized ionized finger image as depicted by the ClearView system.

The ClearView system consists of a camera that lies under a glass electrode. When a finger is placed on the glass electrode of the scanner, a high frequency voltage impulse is applied under the glass and detects the patterns of biophotons emitted from the body through the fingers. The high voltage impulse generates a localized electromagnetic field around the finger thus exciting and amplifying the biophotonic field within the skin, tissue, and nerves within the fingertip. This combination leads to an excitation of the local air molecules, forming room temperature plasma. The energy of the plasma is released via the ionization of the local air molecules, thus emitting photons within the ultraviolet and visible light spectrum. The ionization is captured by the camera and the captured image is relayed into a computer system where the light image is analyzed via the proprietary software to assess it for specific patterns (Fig. 3). Algorithms built upon a large database of data correlated with specific diseases and states of health create a prioritizing scoring method to recognize electrophysiological activity associated with specific organ systems and disease states. At the completion of the finger scans, a ClearView summary score for the cardiovascular system of the body, namely “cardiovascular score”, is produced along with four summary scores for hepatic, renal, respiratory, and gastrointestinal systems in the body. The ClearView system was utilized in this study to determine if it could detect and associate cardiovascular disease with the ClearView cardiovascular score as compared to a medical practitioner's diagnosis

## 2 Methods

### 2.1 Subjects

We conducted a prospective, single center, non-randomized trial of 353 patients who met eligibility criteria (NCT01476995). Men and women aged 18–85 who presented with one of following qualifying diagnoses of coronary artery disease (CAD), gastrointestinal disease, diabetes (type 1 or 2), asthma, COPD, acute or renal failure, acute or chronic hepatitis, or cirrhosis and were a patient of record at the Greater Baltimore Medical Center (GBMC) were enrolled and labeled as the “diagnoses group”. Subjects were only enrolled if they presented with at least one of the confirmed active medical diagnosis:

1. *Cardiovascular system*: coronary artery disease, left sided congestive heart failure with ejection fraction <50%, valvular heart disease, atrial fibrillation, hypertension.
2. *Gastrointestinal/endocrine*: inflammatory bowel disease (including Crohn's disease, ulcerative colitis, or diverticulitis), peptic ulcer disease, IBS, cholecystitis, pancreatitis, malabsorption disorders (including Celiac Sprue), diabetes (Type 1 and Type 2).
3. *Respiratory*: asthma, COPD, bronchitis, emphysema, or pneumonia.
4. *Renal***:** pyelonephritis, acute renal failure, chronic renal failure stages II–V.
5. *Hepatic***:** viral hepatitis, alcoholic hepatitis, steatohepatitis, cirrhosis.

The control group consisted of men and women aged 18–85 who were disease naïve (*N*=64). All vulnerable subjects were excluded. This study was conducted according to US and international standards of Good Clinical Practice (21 CFR part 812 and International Conference on Harmonization guidelines), applicable government regulations and Institutional research policies and procedures, and adherence to the Declaration of Helsinki. All qualifying subjects were consented properly (21 CFR part 50) and medical history and self-reported questionnaires were collected. Additionally, for all subjects enrolled in the qualifying diagnoses groups, all tests and diagnoses from the past six months were obtained from each subject's medical chart by the clinical coordinator. Both groups were scanned with the ClearView scanner by a masked research assistant. This study was ethnically diverse. This study was approved by the local Institutional Review Board at the Greater Baltimore Medical Center (IRB #10-033-05).

### 2.2 Blinding

This was a non-randomized study design. The researcher and research assistant assigned to the study were masked to the patient's diagnoses and documentation. The research assistant informed physicians and staff members that he/she must remain blinded to the diagnoses of the patient but may coordinate with physicians or staff members to schedule subjects for measurement. Subjects were asked whether they would give their permission for the clinical coordinator, who will be collecting medical history information, to contact them directly for scheduling purposes. Eligibility for the control group (healthy individuals identified from a similar population with respect to factors such as age and geographic area) was identified by interviews or pre-screening medical charts, if available. In order to ensure consistency of medical background for both the qualifying diagnoses group and the control group, all efforts were made to standardize the medical history data gathering between the two subject groups (diagnoses group and control group).

### 2.3 Measurement procedures

Participation in the trial required approximately one hour of each subject's time, including obtaining informed consent, answering questions, and image capture. Measurement sessions with the ClearView scanner took approximately fifteen (15) minutes. During measurement sessions, subjects were asked to place each of their ten (10) fingertips on the ClearView glass electrode, and an image was captured through use of the ClearView software. All ten (10) fingers were measured twice; once with a capacitive barrier and once without a capacitive barrier, for a total of twenty (20) images. The capacitive barrier is designed to separate physical and psychological factors of the images. If the device operator deemed that an image was of poor quality (e.g., the subject rolled his or her finger, ambient light entered the image), the image of that finger was retaken. To assess the reproducibility and variability of the measurements, a second measurement session was done 3–5 min after the first one was completed. Thus, each subject had a total of four (4) ClearView measurements, two with and two without capacitive barriers. Each fingertip measurement was about 0.5 s in duration. The first set of measurements was used for the primary analysis endpoints.

### 2.4 Definition of cardiovascular disease

Subjects who were classified under cardiovascular disease presented or were diagnosed by a medical practitioner at GBMC with either coronary artery disease (*N*=72), congestive heart failure (*N*=29), hypertension (*N*=158), atrial fibrillation (*N*=31), and/or valvular heart disease (*N*=16). One hundred ninety-five (195) subjects were diagnosed having at least one cardiovascular disorder based on physical symptoms, risk factors, physical exam, and results from procedures, blood work and diagnostics.

### 2.5 Statistical analyses

The ClearView system provides a score associated with the cardiovascular system on a continuous scale from 0 to 5 representing the functionality of the system from good to poor. The primary hypothesis of this study was there would be an association of the ClearView cardiovascular score with the presence or absence of cardiovascular disease in an active medical diagnosis, quantified by an odds ratio. This trial had a planned sample size of at least 360 subjects. With an expected odds ratio of 1.2, a two-sided significance level of *α*=0.05, 360 subjects would yield at least 95% power for each of the primary analyses. Sample size calculations were conducted using SAS 9.4 PROC POWER (SAS Institute, Cary NC).

Baseline characteristics, including age, sex, and other chronic conditions, were compared between cardiovascular subjects and controls. Logistic regression model was fitted to examine the association between the ClearView cardiovascular score and the presence of cardiovascular disease adjusting for potential confounders, age, and diagnoses of chronic conditions related to liver, kidney, respiratory system, and gastrointestinal/endocrine deficiency. The effect modification by sex was examined using stratified analysis, although no effect modifications by the diagnoses of liver, kidney, respiratory, and gastrointestinal disorders were assumed.

In all models, odds ratios and areas under the receiver operating characteristic (ROC) curves (AUC) with 95% confidence intervals were summarized on measurements with and without capacitive barrier. All analyses were conducted in SAS 9.4.

## 3 Results

In total, 195 subjects had at least one cardiovascular diagnosis and 64 were completely healthy subjects, giving a sample of 259 individuals for the analysis (Table 1). Among the 64 controls, 25 (39.06%) were men. Among the 195 subjects with cardiovascular diagnosis, 113 (57.95%) were men. The mean age of cardiovascular subjects was 64.22 (95%CI: 62.44, 65.99) and the mean age of controls was 44.14 (95%CI: 40.73, 47.55). Table 2 summarized the distribution of clinical comorbidities of the 195 subjects with a cardiovascular diagnosis and ClearView system scores in the sample.

As shown in Table 1, the majority of subjects with cardiovascular diagnosis had hypertension, followed by Coronary Heart Disease. As illustrated in Table 2, almost half of the subjects with cardiovascular problems had gastrointestinal disorders diagnosed, among which 74 were diabetics. The proportion of respiratory diagnosis in the 195 subjects with cardiovascular problem was 37.45%, the majority of which were asthma or COPD. Chronic renal failure accounts for the highest proportion of renal problems among subjects with cardiovascular diagnosis. The major liver disorder among the same group was viral hepatitis.

In Table 3, the overall odds ratios of the cardiovascular score to predict the overall diagnosis of cardiovascular disease were 4.03 (95%CI: 2.71, 6.00) and 2.84 (95%CI: 1.98, 4.09) for measures completed with and without the capacitive barrier, respectively. This indicated a substantial increase in the likelihood of an electrophysiological disturbance within the cardiovascular system for each unit increase of the ClearView score. For each cardiovascular disorder, the odds ratios ranged from 2.39 to 5.43 for measurements with capacitive barrier and almost all AUCs were greater than 0.80 except for valvular heart disease. In the absence of the capacitive barrier, the odds ratios ranged between 2.84 and 4.44 and all AUCs were greater than 0.70.

Potential confounding effects by age and diagnosis of other co-morbidities were summarized in Table 4. The odds ratios adjusted by age did not change much compared to those in Table 3, although AUCs increased substantially by adding age in the logistic regression model. Adding other diagnosis of chronic diseases to the logistic regression model, however, did not alter odds ratios or AUCs much when age had been adjusted. This indicated that the diagnosis of other chronic diseases was not a confounder for the association between ClearView score and the diagnosis of cardiovascular disease. In addition, the diagnoses of other chronic diseases were not significant predictors for cardiovascular disease when ClearView score and age were in the model. In all adjusted models, odds ratios and AUCs for measurements with capacitive barrier were consistently higher than those without capacitive barrier, although the differences were relatively small.

The odds ratios by sex were higher than the crude odds ratio, indicating an effect modification by sex, when capacitive barriers were used in finger scans. In contrast, the odds ratios by sex for measurements without capacitive barrier did not differ much from the unstratified odds ratio. The odds ratios and AUCs for measurements with capacitive barrier were higher than those without capacitive barrier.

## 4 Discussion

In this project, the ability of the ClearView system to detect presence or absence of disease demonstrated strong association with the diagnosis of cardiovascular disease, adjusting for age yet independent of other comorbidities. In other words, the high discrimination ability on disease and healthy subjects found in the ClearView system's indication of presence or absence of disease did not depend on the presence of other comorbidities. The presence of age, however, contributed significantly to the prediction of cardiovascular disease, which was consistent with results found in the literature [20].

Limitations included sample size. Due to sample size constraints, the association between the ClearView system and each cardiovascular condition was not investigated when assessing the confounding effects of other chronic conditions. Such associations can be examined in further studies of the ClearView technology. Similarly, the confounding effects of other chronic conditions were assessed as overall diagnoses.

## 5 Conclusions

This study demonstrated the ClearView system's ability to detect the presence or absence of cardiovascular disease. It has also been proven to be a quantitative assessment tool consistent with known behavior of biophoton research done in-vitro. Study findings were consistent across several other diagnosis groups, for measurements both with and without the capacitive barrier (results will be reported in future publications). The device is applicable to a broad spectrum of individuals, as reflected by this study's population. This study incorporated a variety of diagnoses, including long-term disease such as non-communicable diseases (e.g., CVD), as well as subjects who were experiencing more immediate medical issues and required in-patient care. Furthermore, with subjects covering an age range from 18 to 85 years, and an almost equal distribution of men and women, the study population reflected the wide applicability of the ClearView system. Besides determining the ability to detect and diagnose different components of cardiovascular disease, ClearView also demonstrated safety. No serious adverse events were reported or occurred during the study. Out of 353 subjects, each completing multiple measurements on each of their 10 fingers, only three reports of tingling at the fingertips (an anticipated event) were reported. The multiple measurements per subject are indicative of the ability to use the device repeatedly without negative effects. Additionally, there were no reports of any device malfunction during the course of the study.

Our findings suggest that an objective, non-invasive measurement tool could be implemented as a universal screening and treatment efficacy device for CVD. Typically, age, family history or risk factors that increase an individual's risk of developing cardiovascular disease will determine who should be assessed for cardiovascular health risk. Even if individuals may have a low risk of developing CVD, literature indicates there is high likelihood of developing a variety of cardiovascular issues of varying severity, and therefore may not be properly assessed, diagnosed, or treated. By screening patients early, CVD can be prevented and treated properly. Implementation of the ClearView system could not only consistently identify patients that could be at risk early, but could effectively reduce the national prevalence of disease as well as decrease the mortality rate associated with CVD. Through early detection, CVD could be prevented or treated properly which would not only ameliorate the risk factors associated with the disease, but could cut medical expenditures and all around health care costs. In today's current cardiovascular disease diagnosis model, patients may undergo a myriad of tests depending on symptomology and severity of early results. Various tests are utilized to make a true diagnosis and can take weeks even months to complete all appropriate testing and even longer to make a definitive diagnosis. Early screening usually involves blood tests and an electrocardiogram (ECG) and based on these results, further testing may or may not be required. Typically echocardiograms, stress tests, cardiac computerized tomography (CT) scan, magnetic resonance imaging (MRI), coronary angiogram and even a myocardial biopsy may be ordered and utilized to rule out or to make an appropriate specific cardiovascular related diagnosis. Although testing for cardiovascular disease has been known to be effective and treatment depending on diagnosis can be highly efficacious, a more effective, less time consuming, more integrated, less invasive model needs to be implemented. Changes in medical care expenditures through early detection and diagnosis will be beneficial for not only patients, but for health insurers, employers, and health care administration. The value of having such an objective screening tool could also assist a medical practitioner in proper and immediate treatment. Providing a physician with an integrated complex aggregate of medical information that can be utilized in real time, will not only allow a provider to make a fully informed decision based on early results, but can optimize health care and assist as an efficient approach to next steps in the standard of care process. Although the ClearView system does not dictate the standard of care process that needs to be followed, by utilizing such an integrative device can reduce the time and amount of tests needed to make an appropriate diagnosis, which in turn can not only cut medical costs, but can also allow patients to be diagnosed in a safe, timely, non-invasive fashion.

## Funding

This work was supported and funded by EPIC Research & Diagnostics, Inc..

## Disclosures

Dr. Nancy R. Rizzo is the CEO, Founder, Chief Science Officer, Shareholder and Chairman of the Board for EPIC Research & Diagnostics, Inc. Co-authors Nicole C. Hank and Jian Zhang are employees of EPIC Research & Diagnostics, Inc.

## Figures and Tables

**Table 1:** Cardiovascular diagnosis' as defined by standard of care and physician diagnosis.

**Cardiovascular disease classification**	Cardiovascular (*N*=195)	Control (*N*=64)
Coronary Heart Disease	72 (36.92%)	0
Left-sided Congestive Heart Failure w/ ejection fraction <50%	29 (14.87%)	0
Valvular heart disease	16 (8.21%)	0
Atrial fibrillation (AF)	31 (15.90%)	0
Hypertension	158 (81.03%)	0

**Table 2:** Sample characteristics comparing cardiovascular subjects with comorbities and control (*N*=259).

**Diagnosis of comorbidities**			Cardiovascular (*N*=195)	Control (*N*=64)
Gastrointestinal/endocrine diagnosis		Yes	97 (49.74%)	0
No	98 (50.26%)	64 (100%)
Gastrointestinal/endocrine diagnosis		Inflammatory bowel disease (IBD)	1 (0.51%)	0
Crohn's disease	5 (2.56%)	0
Ulcerative colitis	4 (2.05%)	0
Diverticulitis	8 (4.10%)	0
Peptic ulcer disease	5 (2.56%)	0
Irritable bowel syndrome	5 (2.56%)	0
Cholecystitis	2 (1.03%)	0
Malabsorption disorders	2 (1.03%)	0
Pancreatitis	7 (3.59%)	0
Diabetes	74 (37.95%)	0
Cardiovascular diagnosis		Yes	195 (100%)	0
No	0 (0%)	64 (100%)
Cardiovascular		Coronary Heart Disease	72 (36.92%)	0
Left-sided Congestive Heart Failure w/ejection fraction <50%	29 (14.87%)	0
Valvular heart disease	16 (8.21%)	0
Atrial fibrillation (AF)	31 (15.90%)	0
Hypertension	158 (81.03%)	0
Kidney diagnosis		Yes	52 (26.67%)	0
No	143 (73.33%)	64 (100%)
Kidney		Pyelonephritis	4 (2.05%)	0
Acute renal failure	18 (9.23%)	0
Chronic renal failure stage II–IV	41 (21.03%)	0
Liver diagnosis		Yes	21 (10.77%)	0
No	174 (89.23%)	64 (100%)
Liver		Viral hepatitis	17 (8.72%)	0
Alcoholic hepatitis	2 (1.03%)	0
Steatohepatitis	2 (1.03%)	0
Cirrhosis	4 (2.05%)	0
Respiratory diagnosis		Yes	73 (37.45%)	0
No	122 (62.56%)	64 (100%)
Respiratory		Asthma	30 (15.38%)	0
Chronic obstructive pulmonary disease (COPD)	37 (18.97%)	0
Bronchitis	8 (4.10%)	0
Emphysema	6 (3.08%)	0
Pneumonia	16 (8.21%)	0
Autonomic (ClearView system scores)	Gastrointestinal score	Mean (s.d.)	2.51 (0.61)	2.23 (0.43)
Cardiovascular score	Mean (s.d.)	3.58 (0.96)	2.72 (0.75)
Respiratory score	Mean (s.d.)	2.15 (0.68)	1.61 (0.44)
Liver score	Mean (s.d.)	0.61 (0.11)	0.60 (0.17)
Kidney score	Mean (s.d.)	1.18 (0.68)	0.82 (0.46)
Physical (ClearView system scores)	Gastrointestinal score	Mean (s.d.)	2.55 (0.63)	1.93 (0.42)
Cardiovascular score	Mean (s.d.)	3.62 (0.88)	2.45 (0.88)
Respiratory score	Mean (s.d.)	2.23 (0.79)	1.34 (0.48)
Liver score	Mean (s.d.)	0.65 (0.27)	0.46 (0.20)
Kidney score	Mean (s.d.)	1.22 (0.62)	0.60 (0.31)

**Table 3:** Unadjusted association between ClearView cardiovascular score and diagnoses of cardiovascular disorders.

				**With capacitive barrier**		**Without capacitive barrier**	
**ClearView response**	**Qualifying diagnosis**[1]	**Qualifying diagnosis *N***	**Control *N***	**Odds ratio for having disease**[2]**(95% Wald CI)**	**AUC (95%CI)**	**Odds ratio for having disease**[2] **(95% Wald CI)**	**AUC (95%CI)**
Cardiovascular score	Cardiovascular	195	64	4.03*** (2.71, 6.00)	0.83 (0.77, 0.88)	2.84*** (1.98, 4.09)	0.75 (0.69, 0.81)
Cardiovascular score	Coronary artery disease	72	64	4.36*** (2.61, 7.30)	0.83 (0.76, 0.90)	3.18*** (2.02, 5.0)	0.77 (0.69, 0.85)
Left-sided congestive heart failure (CHF) with ejection fraction (EF) <50%	29	64	5.43*** (2.70, 10.91)	0.86 (0.78, 0.95)	4.44*** (2.36, 8.35)	0.82 (0.71, 0.93)
Valvular heart disease	16	64	2.39** (1.29, 4.45)	0.72 (0.54, 0.89)	2.86 (1.41, 5.79)	0.72 (0.55, 0.88)
Atrial fibrillation (AF)	31	64	4.73*** (2.46, 9.10)	0.84 (0.76, 0.93)	3.00*** (1.74, 5.16)	0.74 (0.63, 0.86)
Hypertension	158	64	4.41*** (2.87, 6.77)	0.84 (0.78, 0.89)	2.84*** (1.96, 4.11)	0.75 (0.68, 0.82)

*** *p*-value<0.001.

** *p*-value<0.01.

**Table 4:** Associations between ClearView cardiovascular scores and diagnosis of cardiovascular disease, stratified by sex, adjusted for age, and diagnosis of hepatic, renal, gastrointestinal, and respiratory disorders.

				With capacitive barrier		Without capacitive barrier	
ClearView response	Potential con-founders	Cardio-vascular Diagnosis N	Control *N*	Odds ratio (95% CI)	AUC (95%CI)	Odds ratio (95% CI)	AUC (95%CI)
Cardio-vascular score	Age	195	64	3.44*** (2.13, 5.55)	0.91 (0.87, 0.95)	2.15** (1.42, 3.23)	0.88 (0.84, 0.93)
Cardio-vascular score	Age, hepatic	195	64	3.69*** (2.22, 6.14)	0.93 (0.89, 0.96)	2.27*** (1.46, 3.51)	0.90 (0.86, 0.94)
Age, renal	195	64	2.89*** (1.78, 4.71)	0.92 (0.89, 0.96)	1.99** (1.29, 3.09)	0.91 (0.87, 0.94)
Age, gastro-intestinal	195	64	3.57*** (2.02, 6.28)	0.96 (0.93, 0.98)	2.93*** (1.69, 5.06)	0.95 (0.92, 0.97)
Age, respiratory	195	64	2.64*** (1.62, 4.28)	0.93 (0.90, 0.96)	1.80* (1.12, 2.87)	0.92 (0.89, 0.95)
Cardio-vascular score^a^	Male	113	25	5.91*** (2.35, 14.85)	0.93 (0.88, 0.99)	2.88** (1.38, 6.01)	0.92 (0.86, 0.97)
Female	82	39	3.50*** (1.86, 6.59)	0.90 (0.85, 0.96)	2.09** (1.20, 3.64)	0.86 (0.79, 0.92)

*** *p*-value<0.001.

** *p*-value<0.01.

* *p*-value<0.05.

a Adjust for age.
